# A Novel Modelling Approach for Predicting Forest Growth and Yield under Climate Change

**DOI:** 10.1371/journal.pone.0132066

**Published:** 2015-07-14

**Authors:** M. Irfan Ashraf, Fan-Rui Meng, Charles P.-A. Bourque, David A. MacLean

**Affiliations:** 1 Faculty of Forestry, Range Management, & Wildlife, Arid Agriculture University, Murree Road, Rawalpindi, 46300, Pakistan; 2 Faculty of Forestry and Environmental Management, University of New Brunswick, Fredericton, NB, E3B 5A3, Canada; DOE Pacific Northwest National Laboratory, UNITED STATES

## Abstract

Global climate is changing due to increasing anthropogenic emissions of greenhouse gases. Forest managers need growth and yield models that can be used to predict future forest dynamics during the transition period of present-day forests under a changing climatic regime. In this study, we developed a forest growth and yield model that can be used to predict individual-tree growth under current and projected future climatic conditions. The model was constructed by integrating historical tree growth records with predictions from an ecological process-based model using neural networks. The new model predicts basal area (BA) and volume growth for individual trees in pure or mixed species forests. For model development, tree-growth data under current climatic conditions were obtained using over 3000 permanent sample plots from the Province of Nova Scotia, Canada. Data to reflect tree growth under a changing climatic regime were projected with JABOWA-3 (an ecological process-based model). Model validation with designated data produced model efficiencies of 0.82 and 0.89 in predicting individual-tree BA and volume growth. Model efficiency is a relative index of model performance, where 1 indicates an ideal fit, while values lower than zero means the predictions are no better than the average of the observations. Overall mean prediction error (BIAS) of basal area and volume growth predictions was nominal (i.e., for BA: -0.0177 cm^2^ 5-year^-1^ and volume: 0.0008 m^3^ 5-year^-1^). Model variability described by root mean squared error (RMSE) in basal area prediction was 40.53 cm^2^ 5-year^-1^ and 0.0393 m^3^ 5-year^-1^ in volume prediction. The new modelling approach has potential to reduce uncertainties in growth and yield predictions under different climate change scenarios. This novel approach provides an avenue for forest managers to generate required information for the management of forests in transitional periods of climate change. Artificial intelligence technology has substantial potential in forest modelling.

## Introduction

Climate change has become an important issue of the 21^st^ century. Based on many years of study, the scientific community has concluded that changes in global climate are mainly caused by increases in anthropogenic emissions of greenhouse gases to the atmosphere [1]. Evidence from many sources indicates that climate change is getting stronger and this upward trend is expected to amplify into the future [2,3]. Global climate models (GCMs) predict that there will be significant change in climate by the end of the current century. For the Maritime Provinces of eastern Canada, GCMs predict a 2–4°C temperature increase during summer and a 1.5–6°C increase in winter [4].

Climatic factors have a direct impact on tree growth and overall forest productivity, as well as indirect impacts on forest ecosystem functioning [5,6]. Climate change will modify tree-growing environments by altering site conditions, such as soil water content, atmospheric humidity, soil and air temperatures, and the length of growing seasons. The forest industry is expected to be the most affected by climate change, compared to other resource-based industries. This is because normal harvesting rotations for timber range from 50 to 100 years for most tree species and present-day forests are expected to experience a period of transition from current to future growing conditions over the next 100 years. Forests provide important socio-economic and ecological services, and existing forests cannot be replaced quickly over the short term to accommodate the anticipated changes in tree-growing environment. Therefore, existing forests need to be managed during this period of transition with well-informed management plans that account for the effects of climate change.

The quality of forest management plans hinges on the quality and reliability of growth and yield (G & Y) predictions. Traditionally, G&Y models are developed from large amounts of historical data using statistical techniques [7,8]. These models usually require simple inputs and predict with relatively low bias at regional scales [9,10]. Growth models are available for even-aged and uneven-aged stands as well as single and mixed species forests. These models have the capability to produce information from individual-tree to stand and forest levels. However, these models are empirical in nature and as a result are applicable only to stand growing conditions similar to the conditions for which they were developed [10,11]. Also, these empirical models rely on a core assumption that climatic and environmental conditions significant to tree growth are not violated [10,12,13]. It has been realised by scientists that G&Y predictions using traditional empirical models could be biased under a changing climatic regime [14–17].

Process-based models are widely used to investigate forest response to climatic change because they predict tree growth based on biological cause-and-effect associations [12,14,18,19]. Gap models are a special type of process-based forest ecosystem models that can take into account climatic and environmental influences based on eco-physiological principles [20]. Gap models are valuable tools to study tree growth, species composition, and stand-structure dynamics under diverse climatic conditions [21,22]. Considerable attempts had been made to use gap models to evaluate climate change impacts on forests [23–29]. However, gap models have rarely been used in conventional forest management planning due to their intensive data requirements, model structure complexity, and incompatibility with management needs to produce G&Y information [8,30]. Process-based models do not have the same accuracy at regional scales compared to conventional models (empirical), because they are not developed to account for site differences across regions.

There is an urgent need for practical and simple G&Y models, easily operated by forest managers, and yet sophisticated enough to predict future forest dynamics under a changing climate. The preferred models should be based on empirical data, but should not be as complicated as process-based models. The objective of this study was to develop a simple G&Y model that (1) utilises conventional tree- and stand-growth attributes, and (2) has the capability to predict individual-tree growth under different climate-change scenarios. The new model was developed by combining tree growth information generated with a process-based model and historical data acquired from permanent sample plots (PSPs).

## Materials and Methods

### 2.1. Modelling approach

Artificial intelligence technology was employed in this study. This growing technology has a potential to solve complex problems in simulation studies. The technology was used to develop a simple G&Y model for prediction under conditions of changing climate by integrating historical tree records and data from an ecological process-based model. Historical tree records were extracted from provincial PSP datasets of the Nova Scotia Department of Natural Resources (NS DNR). A process-based gap model (i.e., JABOWA-3) was used to project tree growth under three climate-change scenarios [29]. This model simulates tree growth based on key assumptions: i.e. (1) trees grow at a maximum rate under optimal conditions, which is influenced by maximum tree age, diameter, and height; (2) tree growth is controlled by biotic and abiotic factors such as inter-tree competition, temperature, and soil water and nutrient content. JABOWA-3’s detailed climate-related functions make it a useful tool in the study of tree species response to climate change. JABOWA-3 had been previously used for climate-change impact assessments of forests in NS [29]. Historical and projected tree growth data were both designated as target quantities (“observations”) and employed in the construction and validation of independent sub-models of diameter- and height-increment. The sub-models were designed to predict individual-tree basal area (BA) and volume increments. Diameter increment was modeled as BA increment (expressed in cm^2^ 5year^-1^) and height increment as volume-increment (m^3^ 5year^-1^). Tree BA was calculated using standard BA-diameter relations, while volume estimates were based on Honer *et al*. metric volume tables relevant to the study area [31]. Both diameter and height can easily be estimated by a re-arrangement of related equations.

### 2.2. Artificial neural networks

**Fig 1:**
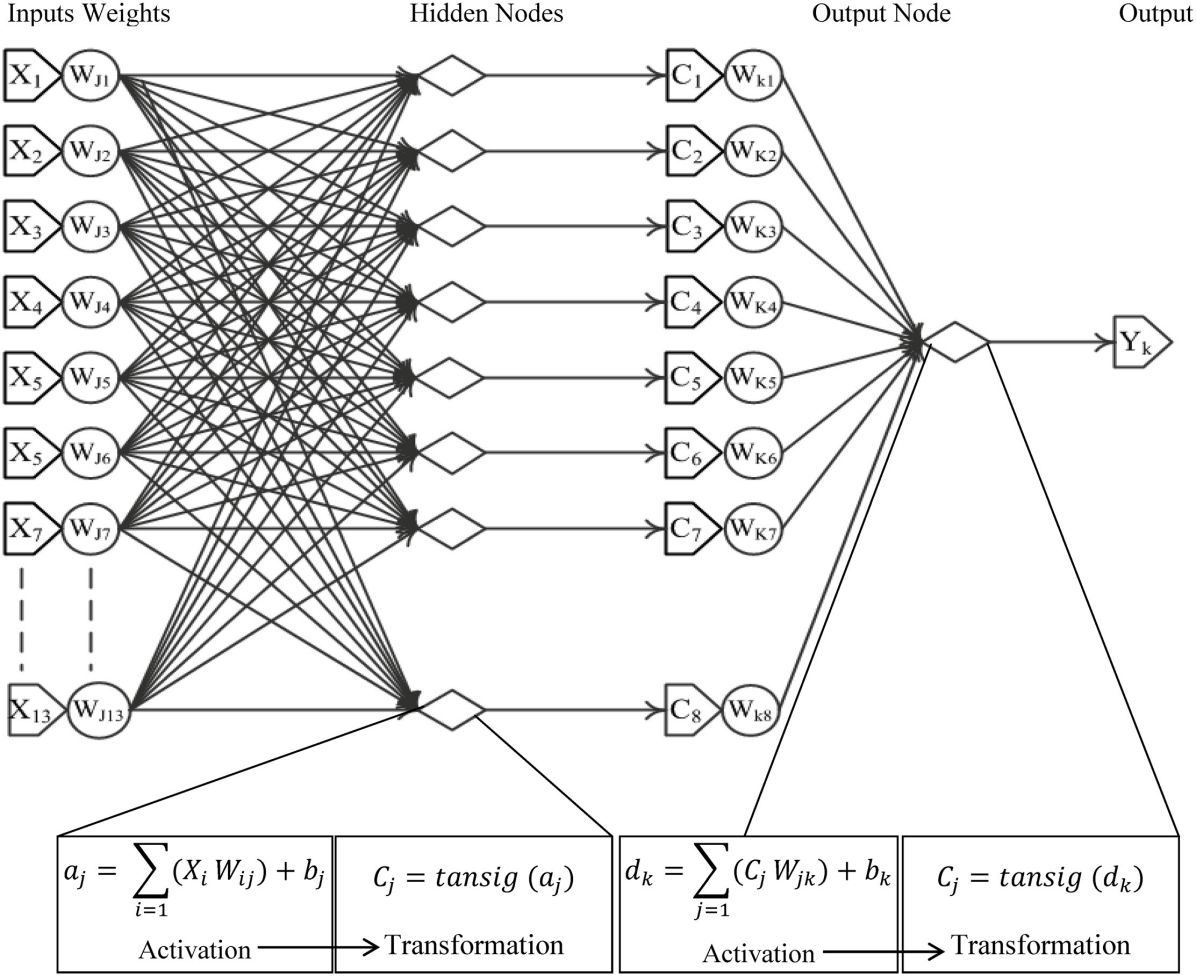
Data flow and ANN structure with eight hidden-layer nodes for the prediction of individual-tree growth.

Artificial neural networks (ANNs) are system-constructs of emerging artificial intelligence technology. They are information-processing systems that are inspired by the functioning of the human brain and have been used to model complex, non-linear interactions in natural systems [32]. A typical ANN consist of inputs (input-layer nodes) and computational units (hidden-layer nodes) connected by weighted links influencing the output calculation [32]. A common form of the three-layer feedforward back-propagation network was used to create independent individual-tree BA and volume increment sub-models. Multi-layer feedforward networks with nonlinear transfer functions were used in this study, because of their ability to discover nonlinear relations between input and output attributes [33]. Thirteen normalised inputs were entered into the network and passed on to the hidden-layer nodes, where a weighted sum of inputs was calculated according to eq (1). The product of this calculation was then passed on to a hyperbolic tangent-sigmoidal transfer function (via eq (2)). Calculations by the hidden-layer nodes were passed forward as input to the output node, with intermediate processing steps described by eqs (3) and (4).

$$a_{j}=\sum_{i=1}(X_{i}\;W_{ij})+b_{j}$$ (1)

$$c_{j}=tansig\left(a_{j}\right)$$ (2)

$$d_{k}=\sum_{j=1}(c_{j}\;W_{jk})+b_{k}$$ (3)

$$y_{k}=tansig\left(d_{k}\right)$$ (4)

where *X* is the input, *Y* the output, *W* the connection weight, and *b* the bias for a given ANN of *i* input, *j* hidden, and *k* output nodes. Variables *a*_*j*_, *c*_*j*_, *d*_*k*_, and *y*_*k*_ are intermediate calculation products. The information flow and structure of ANNs formulated in this study are illustrated in Fig 1Development of ANN models was carried out using MATLAB software (The MathWorks, Inc, USA). MATLAB tools and add-on modules facilitate neural-network-model development and statistical analysis [33]. Computer programme script was developed in default MATLAB editor and files are available from the authors upon request.

#### 2.2.1. Artificial neural network input-layer nodes

At the tree level, individual trees were described in the model by their species and size (i.e., BA). Forty-three tree species present in the PSPs were grouped into eighteen separate species categories for simplification (Table 1; [34]). Dominant tree height was used to reflect stand developmental stage. Total stand BA of softwoods and hardwoods (BASW, BAHW) were used to reflect the overall stand composition. Inter-tree competition was captured by ranking trees according to their size. PSP total BA of all larger softwood and hardwood trees than a given tree (BALSW, BALHW) were used as inputs to account for competition among individual trees [35]. Stocking, a descriptor of tree occupancy of a stand to a standard value of stand-density conditions [36], was selected as a model input to help capture stand-growing conditions. Site conditions and growing environment were defined by biophysical variables, such as (i) incident solar radiation (SR), (ii) cumulative growing degree-days (GDD), and (iii) indices of soil nutrient and water content. Present-day and future forest response to changing climate was defined for four main forest growth scenarios: historical tree-growth records (PSP data) were considered to represent present-day climate, whereas JABOWA-3 generated data for three climate-change scenarios were used to represent future growing conditions. The inputs, their units, abbreviated codes, and species groups are listed in Tables 1 and 2. Further detail about inputs selection and calculation procedures is available in Ashraf *et al*. [34].

**Table 1:** Tree species groups, their codes, and assigned values used as categorical input for ANNs of basal area and volume growth.

**Input Node 9values**	**Code**	**Species Group**	**Common Name**	**Scientific Name**
1	ASH	Ash	Black ash	*Fraxinus nigra*
			White ash	*Fraxinus americana*
2	BE	Beech	American beech	*Fagus grandifolia*
3	BF	Balsam fir	Balsam fir	*Abies balsamea*
4	BS	Black spruce	Black spruce	*Picea mariana*
5	EH	Eastern hemlock	Eastern hemlock	*Tsuga canadensis*
6	MH	Misc. hardwoods	Alder (speckled)	*Alnus rugosa*
			Ailanthus	*Ailanthus altissima*
			Apple	*Malus sp*.
			Black cherry	*Prunus serotina*
			Choke cherry	*Prunus virginiana*
			Dogwood	*Cornus sp*.
			English oak	*Betula populifolia*
			Hawthorn	*Crataegus sp*.
			Ironwood	*Ostrya virginiana*
			Mountain ash	*Sorbus Americana*
			Mountain maple	*Acer spicatum*
			Pin cherry	*Prunus pensylvanica*
			Service berry	*Amelanchier sp*.
			Striped maple	*Acer pensylvanicum*
			American elm	*Ulmus americana*
			Willow	*Salix sp*.
7	MP	Misc. pines	Jack pine	*Pinus banksiana*
			Red pine	*Pinus resinosa*
			Scots pine	*Pinus sylvestris*
8	MS	Misc. softwoods	Japanese larch	*Larix kaempferi*
			Norway spruce	*Picea abies*
			Sitka spruce	*Picea strobus*
9	PO	Poplar	Balsam poplar	*Populus balsamifera*
			Larch	*Larix sp*.
			Trembling aspen	*Populus tremuloides*
			Large-tooth aspen	*Populus grandidentata*
10	RM	Red maple	Red maple	*Acer rubrum*
11	RO	Red oak	Red oak	*Quercus rubra*
12	RS	Red spruce	Red spruce	*Picea rubens*
13	SM	Sugar maple	Sugar maple	*Acer saccharum*
14	TL	Tamarack	European larch	*Larix decidua*
			Tamarack	*Larix laricina*
15	WB	White birch	Grey birch	*Quercus robura*
			White birch	*Betula papyrifera*
16	WP	White pine	White pine	*Pinus strobus*
17	WS	White spruce	White spruce	*Picea glauca*
18	YB	Yellow birch	Yellow birch	*Betula alleghaniensis*

Adapted from *Ashraf et al*. *2013* [34].

**Table 2:** Input variables for ANN-models of basal area (BA) increment and volume increment.

**Input Node #**	**Code**	**Description**	**Units**
1	BA	Basal area	cm^2^
2	BASW	Sum of SW BA in PSP	m^2^ ha^-1^
3	BAHW	Sum of HW BA in PSP	m^2^ ha^-1^
4	BALSW	Sum of BA of larger SW trees ^a^	m^2^ ha^-1^
5	BALHW	Sum of BA of larger HW trees ^a^	m^2^ ha^-1^
6	DOM-HT	Dominant height	m
7	SMO	Soil moisture	1–7 unit less
8	SN	Soil nutrients	5–47 unit less
9	SPE	Species class identification	1–18 ^b^ unit less
10	STOC-FAC	Stocking factor	Relative index
11	GDD	Growing degree days	Temperature index; > 5°C
12	SR	Solar radiation	watt-hours m^-2^
13	Climate	Climate scenarios	1–4 ^c^ unit less

^a^ Sum of the BA of large trees (based on diameter) in a PSP, larger than a given tree.

^b^ A complete list of species groups and assigned values are provided in Table 1.

^c^ 1: present-day climate (PC); projected climate change scenarios: 2: minimal (B1), 3: moderate (A1B), 4: maximum (A2)

SW: softwood trees; HW: hardwood trees.

### 2.3. Study area

**Fig 2:**
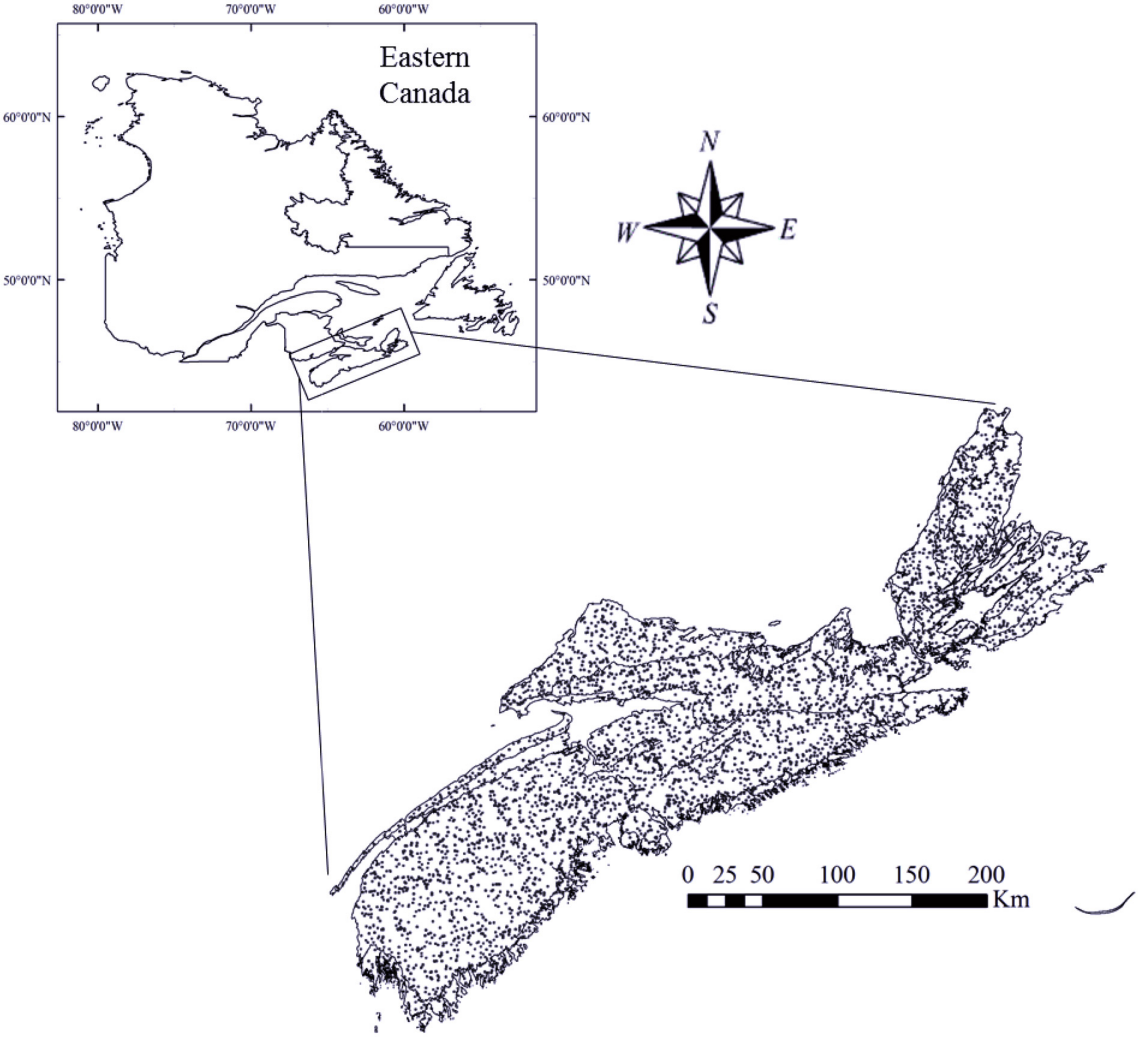
Province of Nova Scotia, Canada, and distribution of permanent sample plots (PSPs).

The Province of NS is located on the east coast of Canada (43°27' N—46° 01' N; 59° 38' W- 66° 16' W). Elevation of the area ranges from 0–540 m above mean sea level. The current climate of the region is temperate humid with an average annual precipitation of 1425 mm along the coast and 1000 mm inland. Average daily air temperature ranges from 16–24°C in summer and around -3°C during winter. Annual GDD (base temperature of 5°C) for the province vary from 1500 to 1750 [36]. Under Canadian-GCM projections, GDDs are expected to increase by about 800–1400 at the end of 21^st^ century. Forests cover 77% of the province, with 50% classified as softwood, 11% as hardwood, 25% as mixedwood, and the remaining 14% as unclassified forests [37]. Major tree species are spruce (*Picea sp*.) at 35% of total wood volume, balsam fir (*Abies balsamea*) at 22%, and broadleaved species at 30% [38]. Location of the study area and the network of PSPs used in this study are indicated in Fig 2.

### 2.4. Data acquisition and preparation

#### 2.4.1. Growth and yield data from permanent sample plots

The required tree and stand information were available from PSP datasets maintained by NS DNR. Permission to use the permanent sample plot data was granted by the NS DNR under a fair-use data agreement with The University of New Brunswick, Canada. Data collection did not involve any destructive sampling of either endangered or protected forest species. The data for this study was obtained from 3250 PSPs established to update forest inventories. Each PSP was of 0.04 ha (fixed size) and circular in shape with a radius of 11.35 m. Measurement of traditional inventory attributes included tree diameter at breast height (DBH) and tree height. Data collection included only trees with a DBH > 9.1 cm. Inventory measurements were collected every five years since ~1960’s, with the most recent data collected in 2006. Analysis of PSP data indicated an average DBH of 15.99 cm (Min = 9.1; Max = 99.6) and an average height of 10.52 m (Min = 0.6; Max = 24.9).

#### 2.4.2. Growth and yield data under climate change

Input data required to predict future forest states with JABOWA-3 includes (i) climatic information, (ii) initial stand conditions, and (iii) site conditions, including soil information. Projected future climate data (climate change) were acquired from Environment Canada [39]. The information related to site conditions was extracted from a digital elevation model (DEM) and GIS thematic maps of NS, while soil-related information was acquired from the Canadian Soil Information Service (CANSIS) [40]. Initial stand conditions were based on PSP records. Tree growth projections were developed for 34 PSP locations across NS, selected to represent nine eco-regions and capture the diverse climatic and site growing conditions of NS. The model was first calibrated using historical weather records and observed PSP data from NS [41]. JABOWA-3 was initiated with existing stand conditions using relevant projected future climates. The model was run 5–7 times for each location to lessen the effect of model stochasticity inherent in model functions of mortality and ingrowth. Individual-tree diameter and height dynamics for the next 90 years were extracted from JABOWA-3 outputs under three projected climate-change scenarios.

#### 2.4.3. Climate change data

Future climate data used for model prediction were derived from the third generation Canadian-GCM (CGCM3-T47) for the fourth assessment period of the IPCC [1]. Climate data produced were based on future emission scenarios of greenhouse gases defined by IPCC. Three illustrative emission scenarios chosen for the study include B1, A1B, and A2 [42]. The emission scenarios were based on future global development with a particular emphasis on greenhouse gases and aerosol-precursor emissions [43]. The A1B scenario was developed to reflect the world in an era of balanced energy use of fossil and non-fossil fuels, rapidly growing economy, and an increasing world population achieving a maximum in the middle of the current century. The B1 scenario reflects an environment friendly world, population trend similar to A1B, and global economy rapidly moving towards a service and information framework with the introduction of clean and efficient energy technologies. The A2 scenario signifies a heterogeneous world, moderate economic growth, and high population growth throughout the century [42]. Ninety year projections of monthly mean temperatures and precipitation (2012–2102) relevant to selected locations of NS were obtained from the CGCM3-T47 simulations for B1, A1B, and A2 climate scenarios. Discrepancy in CGCM3-T47 produced data was calculated by comparing past weather records with the relevant predictions for each location. Monthly mean temperatures were adjusted by accounting for the mean bias, whereas ratio adjustments were used to correct future estimates of precipitation [29]. In this study, we define B1 as a minimal impact, A1B as a moderate impact, and A2 as a maximum impact scenario.

### 2.5. Neural network training and optimum model structure

The dataset assembled for training and validating the individual ANNs for BA and volume increment predictions consisted of 326,395 records of tree growth for each network; 220,657 records extracted from historical datasets (i.e., PSPs) as representative of present-day forest conditions, and 105,738 tree-growth records generated with JABOWA-3 for the three climate scenarios (B1, A1B, and A2). The dataset was then divided into a training sub-dataset (75% of the original dataset) and validation sub-dataset (25%). Both training and validation sub-datasets were normalised to a range from -1 to 1, due to widely varying ranges and magnitudes of inputs and outputs [44].

During training, the ANNs predicting BA and volume increments were compared with their corresponding target values, differences calculated, and propagated back through the network for weight optimisation and error reduction [45–47]. Number of hidden-layer nodes is a sensitive structural parameter in ANNs, as too few can lead to under-fitting of the target data and too many can lead to over-fitting [47]. An early-stop option was used to stop training in order to prevent over-fitting of the target data.

In this study, we examined ANN structures with four to ten hidden nodes and evaluated their prediction accuracy. In ANN training, mean squared error (MSE) describes the variance and bias of the predictor with respect to normalised observation values. Pearson’s correlation coefficient (R) and BIAS (eq (5)) were used to evaluate training accuracy (predicted vs. observed); BIAS demonstrates the average difference between predicted and observed data.

$$BIAS=\;\frac{\sum_{i=1}^{n}\left(O_{i}-S_{i}\right)}{n}$$ (5)

where *O*_*i*_ denotes observed values of tree *i*, *S*_*i*_ is the associated predicted value, and *n* is the number of prediction-to-observation data-point comparisons.

Pearson’s correlation coefficient was calculated for normalised data, while BIAS was calculated from de-normalised data. The final ANNs for BA and volume increments were selected based on training accuracy indicated by R, MSE, and BIAS. The ANNs selected with optimised and set weights were validated against independent datasets.

### 2.6. Model validation

The accuracy of ANNs with respect to predicted BA and volume increments was statistically evaluated by comparing against values from the validation sub-dataset. Statistical indicators used in the assessment of accuracy included (i) root mean squared error (RMSE), (ii) BIAS (eq (5)), and (iii) model efficiency (ME, eq (6)). Model efficiency is a relative index of model performance estimated by directly comparing predicted and observed data [48]. Model efficiencies range from 1 to -∞; 1 represents a perfect fit, while values lower than zero means the predictions are no better than the average of the observations.

$$ME=1-\frac{\sum_{i=1}^{n}{\left(O_{i}-S_{i}\right)}^{2}}{\sum_{i=1}^{n}{\left(O_{i}-{\bar{O}}_{i}\right)}^{2}}$$ (6)

where ${\bar{O}}_{i}$ is the mean observed value, others are same as in eq (5).

Observed and ANN-predicted BA and volume increments were plotted against the 1:1 line. We also plotted residuals to get further insight as to the prediction accuracy of ANNs. The validation dataset along with relevant ANN predictions were categorised according to climate scenario, since the model was developed with a special intention to predict forest response under conditions of climate change. The residuals ([observed increment]-[ANN-predicted increment]) vs. individual-tree size (BA) were plotted according to climate scenario.

## Results

### 3.1. Selection of neural network structure

**Fig 3:**
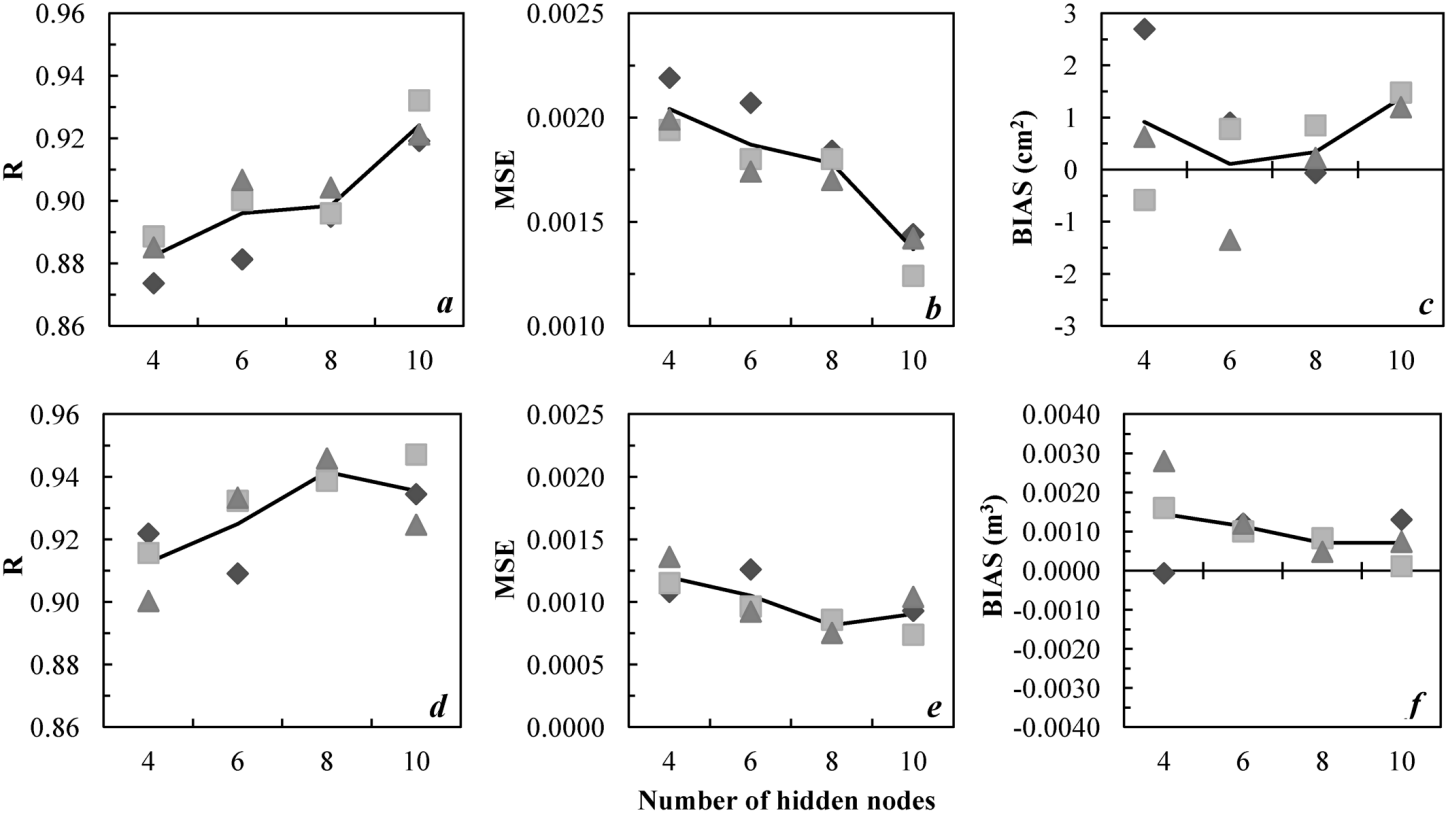
ANN training accuracy for basal area (BA) increment (*a-c*) and volume increment (*d-f*). The points represent results from individual training sessions and the trend lines represent the average of results from the three best models with the same number of hidden-layer nodes. R is the correlation coefficient, MSE the mean squared error of normalised data, and BIAS the average difference between predicted and observed data.

Values of R, MSE, and BIAS for the individual ANNs with four to ten hidden-layer nodes are presented in Table 3; variations according to the number of hidden-layer nodes are illustrated in Fig 3. For BA increment, ANN configurations with four to ten hidden-layer nodes produced R values between 0.87 and 0.93, MSE of 0.00124 to 0.00219, and BIAS of -0.06670 to 2.69360. The R values increased with the number of hidden layer nodes from four to six, but were similar for six and eight hidden layer nodes, and increased with ten hidden nodes (Fig 3*a*). Lowest MSE values were observed for ANNs with ten hidden nodes and greatest with four hidden nodes. BIAS values were consistent, lowest, and close to zero for network structures with eight hidden nodes; BIAS values were progressively higher with four, six, and ten hidden nodes (Fig 3*c*).

**Table 3:** Training accuracy of ANNs for basal area (BA) increment and volume increment with different numbers of hidden-layer nodes.

**Hidden nodes**	**BA increment**			**Volume increment**		
	R	MSE	BIAS	R	MSE	BIAS
4	0.87	0.00219	2.69360	0.92	0.00108	-0.00007
4	0.89	0.00194	-0.58540	0.92	0.00115	0.00160
4	0.89	0.00199	0.62620	0.90	0.00136	0.00280
6	0.88	0.00207	0.89810	0.91	0.00126	0.00120
6	0.90	0.00180	0.77700	0.93	0.00096	0.00100
6	0.91	0.00174	-1.35230	0.93	0.00092	0.00120
8	0.89	0.00184	-0.06670	0.94	0.00083	0.00080
8	0.90	0.00180	0.84440	0.94	0.00086	0.00083
8	0.90	0.00170	0.22040	0.95	0.00075	0.00048
10	0.92	0.00144	1.41510	0.93	0.00093	0.00130
10	0.93	0.00124	1.47580	0.95	0.00074	0.00011
10	0.92	0.00142	1.19360	0.92	0.00104	0.00073

R: correlation coefficient; MSE: mean squared error; both R and MSE are estimated from normalised data ([-1] to [+1]).

BIAS is calculated from denormalised data using eq (6); BA increment (basal area; cm^2^5year^-1^); volume increment (m^3^ 5year^-1^).

ANN prediction accuracy for individual-tree volume increment (m^3^ 5year^-1^) is given as a function of the number of hidden-layer nodes in Fig 3*d*–3*f*. The R values ranged from 0.90 to 0.95 and MSE from 0.00074 to 0.00136, whereas BIAS ranged from -0.00007 to 0.00280. Overall highest R and lowest MSE were produced with ANNs with eight hidden nodes, while average minimum BIAS occurred when eight hidden nodes were used. In general, prediction accuracy increased from four to eight hidden nodes, and decreased from eight to ten nodes.

### 3.2. Model validation

**Fig 4:**
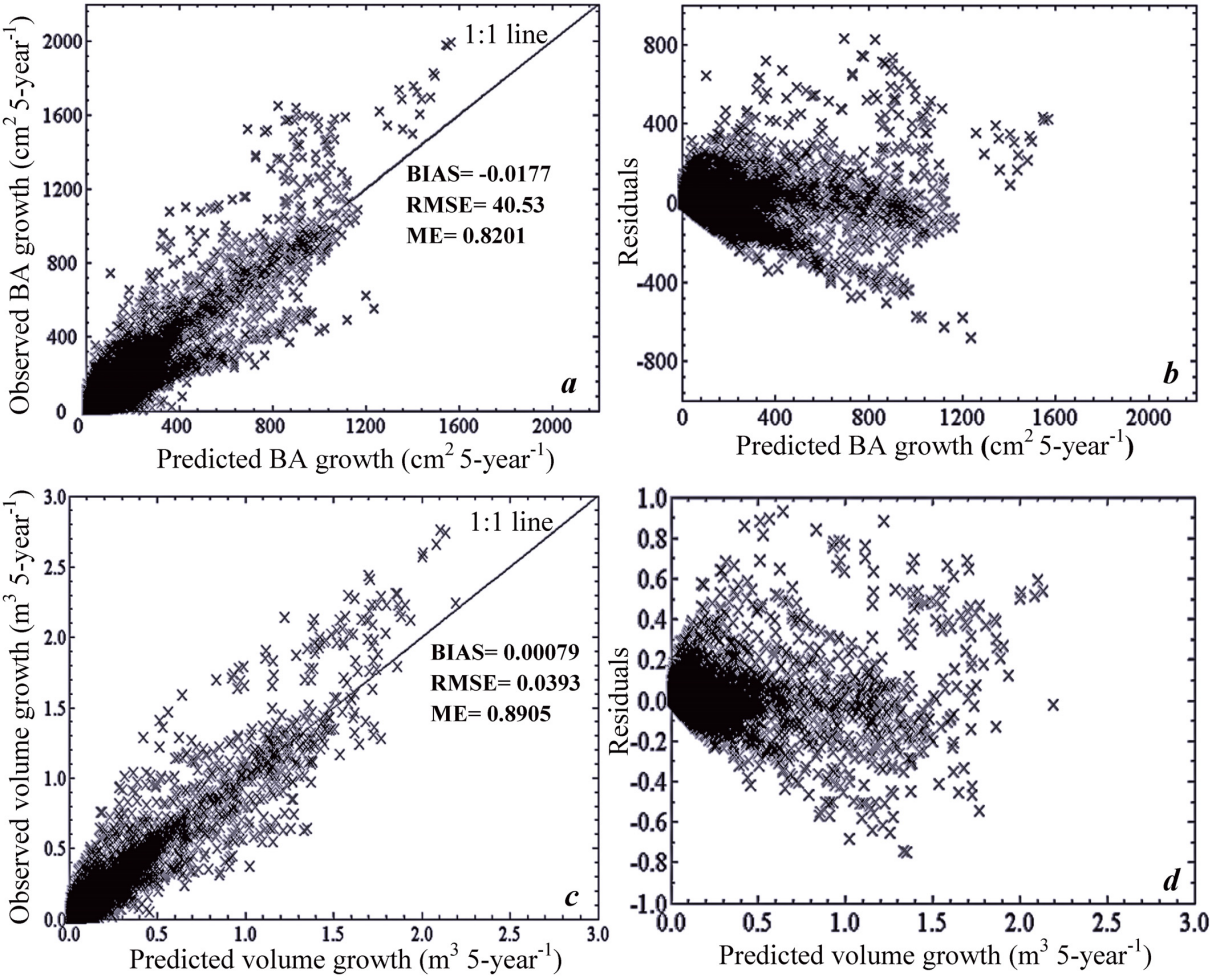
ANN model validation of individual-tree observed and predicted basal area and volume increment. (***a***) basal area increment (BA: cm^2^ 5-year^-1^), (***b***) BA increment residual plot, (***c***) volume increment (m^3^ 5-year^-1^), and (***d***) volume increment residual plot. ME is model efficiency, BIAS is the average difference between predicted and observed data (eq 6), and RMSE is the root mean squared error.

Scatter plots and residuals generated from ANN validation are presented in Fig 4. Predictions with the optimised ANN for BA increment, when compared to the validation data produced a BIAS of -0.0177, RMSE of 40.53, and ME of 0.82. Similarly, ANN-predictions for volume increment generated a BIAS of 0.00079, RMSE of 0.0393, and ME of 0.89. High ME-values (> 0.80) indicated good overall ANN performance for both BA and volume increment under present-day and future projected climates. Overall, average difference between ANN-predicted and observations (i.e., BIAS) was low for both BA and volume increments.

**Fig 5:**
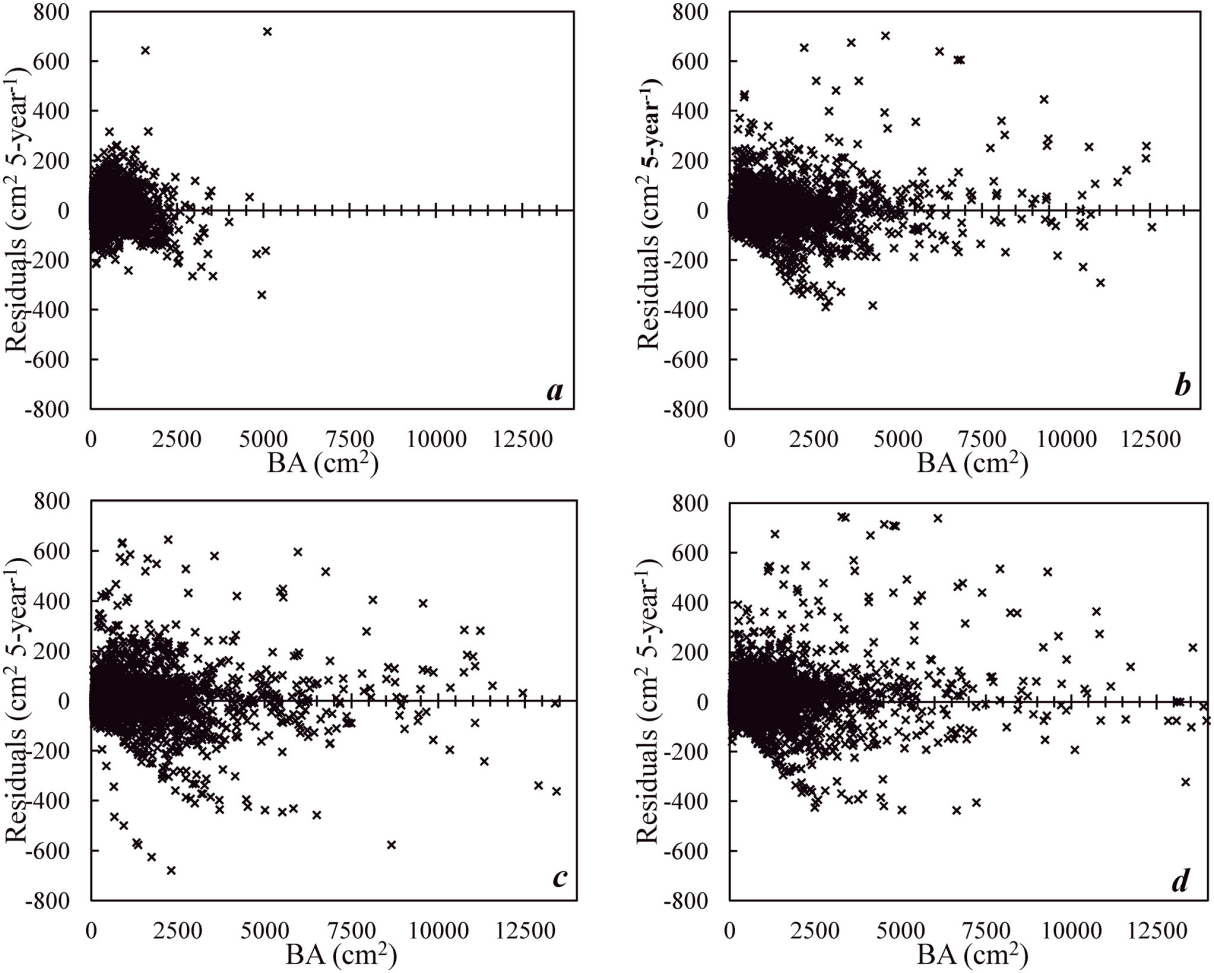
Scatterplots of individual-tree basal area (BA) increment as a function of residuals of BA increment during validation (*a*) present-day climate scenario; (*b*) B1, (*c*) A1B, (*d*) A2 climate-change scenarios. Residuals = (observed)—(ANN-predicted) individual-tree BA increment [cm^2^ 5-year^-1^].

**Fig 6:**
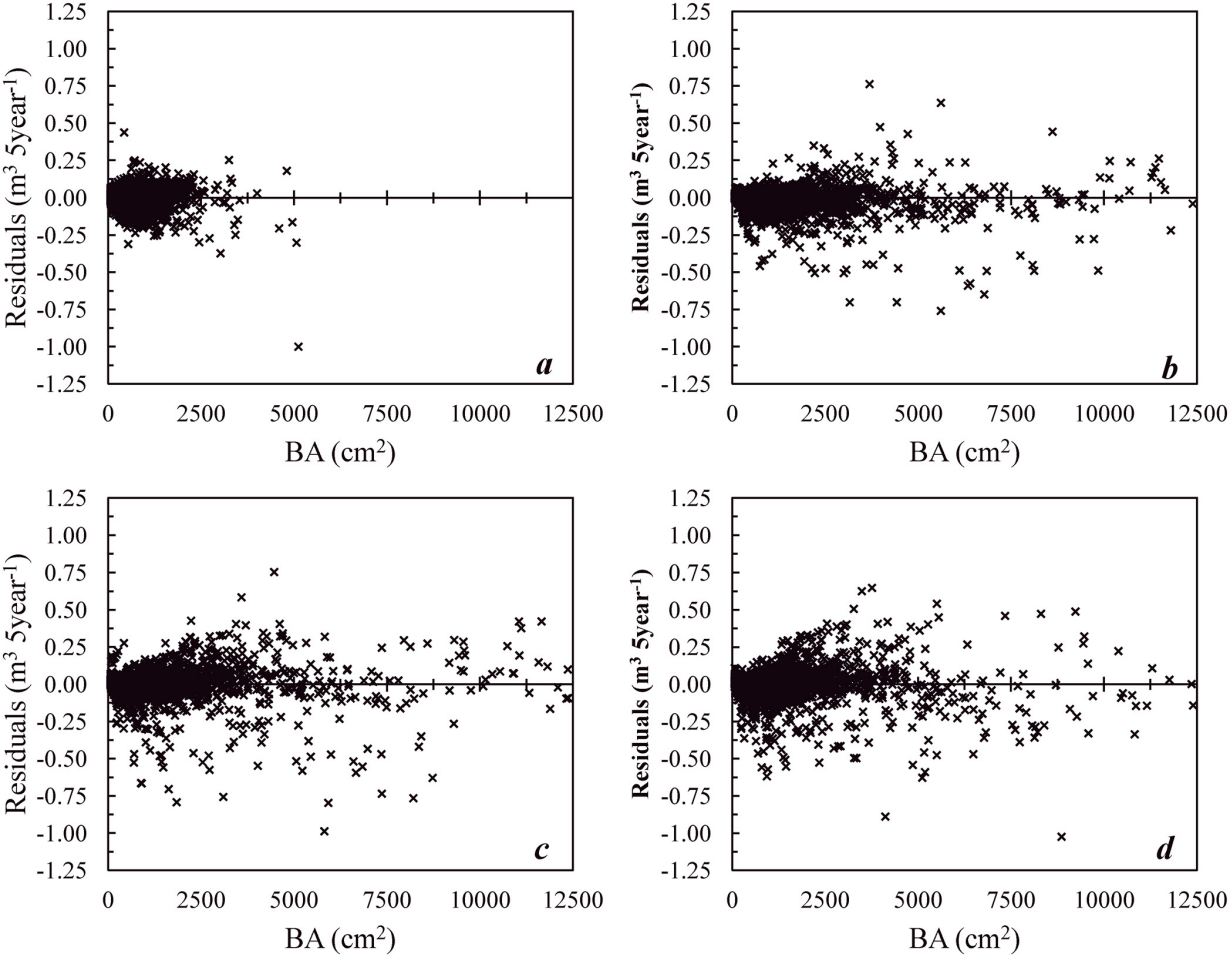
Scatterplots of individual-tree BA increment as a function of residuals of volume increment during validation (*a*) present-day climate scenario; (*b*) B1, (*c*) A1B, (*d*) A2 climate-change scenarios. Residuals = (observed)—(ANN-predicted) individual-tree volume increment [m^3^ 5year^-1^].

Scatter plots of residuals vs. individual-tree BA are presented in Figs 5 and 6.

Most residuals for BA increment were within ± 200 cm^2^ 5-year^-1^ and tree BA was < 2500 cm^2^ (Fig 5*a*).

## Discussion

Many ANNs with random initialisation of weights were trained to avoid the problem of local minima [49,50]. Results presented in Table 3 are the best three runs for each model structure as to individual training accuracies for different number of hidden nodes. With all the runs and model structures examined, the best ANNs for modelling BA and volume increments consisted of eight hidden nodes. Optimised ANNs for both variables consisted of 13 input nodes, eight hidden-layer nodes, and one output node.

There are few related studies available in the literature to compare against, as this is the first time that a G&Y model has been developed and validated against tree data for both present-day (i.e., PSP data) and projected future climates (JABOWA-3 generated data). Statistical indicators of model accuracy (i.e., BIAS, RMSE, and ME) demonstrate good overall model performance compared to conventional models designed for static climatic conditions. The Forest Vegetation Simulator-Inland Empire model (i.e., FVS-IE), generally over-predicted diameter increments by 14% compared to field records, whereas discrepancy in predicting different species diameter ranged from -49.1% to 5.9% [51]. This was reported in a study validating the BA increment component of FVS-IE with forest inventory data across the Inland Empire region, USA. Basal area increment models developed in Norway generated coefficients of determination (r^2^) ranging from 0.26 to 0.55 for different tree species [52]. A study evaluated conventional tree-level models for Finland and obtained overall relative BIAS of 2.3 to -6.0% in the prediction of stand BA [53].

Previous studies modelling individual-tree growth with ANNs based on data from the province of NS, reported a RMSE of 26.14 and 23.50, respectively, and ME of 0.39 and BIAS of 0.1356 for BA increment [34,54]. In general, the model developed in this study demonstrated better overall performance in terms of BIAS and ME compared to past studies in NS, albeit with a slight increase in RMSE for both BA and volume predictions compared to those reported by Ashraf *et al*.[34]. Addition of JABOWA-3 growth data to account for climate change has increased the dispersion in target data. High R and ME values can also be attributed to the addition of JABOWA-3 generated growth data in the training dataset and should not be interpreted that the newly developed ANNs are substantially better than the other models.

There is always a certain level of uncertainty in model predictions as forest G&Y models are abstractions of highly complex, nonlinear systems. It would be unrealistic to expect that a model can replicate all natural variation exhibited by biological systems. Potential sources of model discrepancy in JABOWA-3 calculations may arise from inaccuracies in GCM-produced future climates [55] and climate-response functions in JABOWA-3 [56]. Further discussion about the limitations of gap models is available in published scientific literature [7,21,57].

All residuals were randomly dispersed along the horizontal axis indicating good overall model performance [58]. This demonstrates, with field records spanning ~40 years of present-day climate, that most stands are young to immature (0–40 years) due to intensive harvests in the past [37,59]. However, dispersion of data points along the x-axis (BA) increased in Fig 5*b*–5*d*, since these Figs show results of 90 years growth predicted by JABOWA-3 under climate change scenarios.

The expanded range in some of the residual points in Fig 5*b*–5*d* indicated that ANNs cannot predict a number of data points (tree growth) produced with JABOWA-3 for future climate scenarios. However, most of the other data points (~98%) were within the same range as in Fig 5*a* for existing field records. Residual analysis revealed that most data points (-∞,-300] or [300, ∞) belonged to red maple (*Acer rubrum* L.) with its growth expected to increase with climate change [29]. Similar trends were observed in residual plots (Fig 6) for volume growth predictions. Generally, these results indicated the advantage of pooling data (JABOWA-predicted + PSP tree data) together for joint influence on ANN development. As the number of data points associated with field-measured PSP data was greater than that of the JABOWA predictions, the PSP data may have helped to adjust some over-prediction with red maple under scenarios of climate change.

It is important to mention other approaches such as potential growth surfaces (PGS), climate envelops (CE), and plant-hardiness zones (PHZ) applied to study forest response to climate change [60–64]. These approaches mostly describe species response to climatic variables and ignore biotic factors critical to tree growth, such as inter- and intra-specific competition [60]. Biotic factors may not be so important for studies at larger spatial scales, such as landscape or biomes, but they are particularly vital for stand- and regional-scale studies [63]. In addition, PGS, CE, and PHZ are developed for discrete time intervals, taking into account 30-year climate normals and their modification under projected climate change [60–64]. Therefore, the inferences drawn based on these approaches are for certain time-periods assuming step-wise changes. However, as climate change is a gradual process, information about successional pathways that present-day forests are expected to take during the transition period is greatly desirable [65]. Furthermore, PHZs define the climate ranges (mainly as a function of temperature) where certain plant species can survive based on species geographic presence-absence [66]. PHZs are developed based on species existence and are not true illustrations of species-growth behaviour. Species can survive within defined PHZ ranges, but may not achieve optimal or even normal growth. Numerous researchers have suggested the importance of finding ways to combine empirical and process-based approaches in the development of hybrid models to address forest-growth response with climate change [e.g., 10,16]. In this study, we introduced a modelling approach to improve the scope of current G&Y models by using knowledge acquired with process-based models [15].

Climate change impact assessments always have uncertainty due to the complex and stochastic nature of biological and climate systems [62,63]. Although model parameters need to be fine-tuned, gap models are considered to have the best model structure for assessing impacts of climatic change over the transitional period of present-day forests [29]. Moreover, gap models can be tailored to provide individual-tree data in the same format as provided by PSP records. In this study, projecting tree growth under the influence of climate change with a gap model in developing empirical G&Y models is a unique contribution to forest modelling addressing non-static growing conditions. However, opportunities exist for expanded studies using other types of process-based models and comparative studies of different modelling techniques in assessing forest-related climate change impacts.

## Conclusions

In this study, we developed a simple and practical G&Y model that can predict individual-tree BA increment and volume increment over 5-year intervals. The model is based on ANNs and has the capability to predict growth under current climate conditions as well as under three projected climate-change scenarios. The G&Y model was formulated and validated against over 40 years of tree data from 3250 PSPs, together with 90 years of data for projected future climates. The model predicted individual-tree growth in multi-species forest for both even-aged and uneven-aged stands. The model requires traditional forest inventory attributes, in addition to incident solar radiation, GDDs, and indices of soil nutrient and soil water content at the location of PSPs as primary input. This study demonstrated a novel modelling approach to integrate climate change impacts in forest G&Y models. The study also demonstrated the virtue of using artificial intelligence in modelling forest G&Y.
